# A high-performance deep-learning-based pipeline for whole-brain vasculature segmentation at the capillary resolution

**DOI:** 10.1093/bioinformatics/btad145

**Published:** 2023-03-22

**Authors:** Yuxin Li, Xuhua Liu, Xueyan Jia, Tao Jiang, Jianghao Wu, Qianlong Zhang, Junhuai Li, Xiangning Li, Anan Li

**Affiliations:** Shaanxi Key Laboratory for Network Computing and Security Technology, School of Computer Science and Engineering, Xi’an University of Technology, Xi’an 710048, China; Shaanxi Key Laboratory for Network Computing and Security Technology, School of Computer Science and Engineering, Xi’an University of Technology, Xi’an 710048, China; HUST-Suzhou Institute for Brainsmatics, Suzhou 215123, China; HUST-Suzhou Institute for Brainsmatics, Suzhou 215123, China; Shaanxi Key Laboratory for Network Computing and Security Technology, School of Computer Science and Engineering, Xi’an University of Technology, Xi’an 710048, China; Shaanxi Key Laboratory for Network Computing and Security Technology, School of Computer Science and Engineering, Xi’an University of Technology, Xi’an 710048, China; Shaanxi Key Laboratory for Network Computing and Security Technology, School of Computer Science and Engineering, Xi’an University of Technology, Xi’an 710048, China; Britton Chance Center for Biomedical Photonics, Wuhan National Laboratory for Optoelectronics, MoE Key Laboratory for Biomedical Photonics, Huazhong University of Science and Technology, Wuhan 430074, China; HUST-Suzhou Institute for Brainsmatics, Suzhou 215123, China; Britton Chance Center for Biomedical Photonics, Wuhan National Laboratory for Optoelectronics, MoE Key Laboratory for Biomedical Photonics, Huazhong University of Science and Technology, Wuhan 430074, China; HUST-Suzhou Institute for Brainsmatics, Suzhou 215123, China

## Abstract

**Motivation:**

Reconstructing and analyzing all blood vessels throughout the brain is significant for understanding brain function, revealing the mechanisms of brain disease, and mapping the whole-brain vascular atlas. Vessel segmentation is a fundamental step in reconstruction and analysis. The whole-brain optical microscopic imaging method enables the acquisition of whole-brain vessel images at the capillary resolution. Due to the massive amount of data and the complex vascular features generated by high-resolution whole-brain imaging, achieving rapid and accurate segmentation of whole-brain vasculature becomes a challenge.

**Results:**

We introduce HP-VSP, a high-performance vessel segmentation pipeline based on deep learning. The pipeline consists of three processes: data blocking, block prediction, and block fusion. We used parallel computing to parallelize this pipeline to improve the efficiency of whole-brain vessel segmentation. We also designed a lightweight deep neural network based on multi-resolution vessel feature extraction to segment vessels at different scales throughout the brain accurately. We validated our approach on whole-brain vascular data from three transgenic mice collected by HD-fMOST. The results show that our proposed segmentation network achieves the state-of-the-art level under various evaluation metrics. In contrast, the parameters of the network are only 1% of those of similar networks. The established segmentation pipeline could be used on various computing platforms and complete the whole-brain vessel segmentation in 3 h. We also demonstrated that our pipeline could be applied to the vascular analysis.

**Availability and implementation:**

The dataset is available at http://atlas.brainsmatics.org/a/li2301. The source code is freely available at https://github.com/visionlyx/HP-VSP.

## 1 Introduction

The cerebrovascular is a large and complex network of arteries, veins, and capillaries throughout the brain. It provides nutrients for brain activity and transports metabolic waste (Muoio et al. 2014). The morphological structure and spatial distribution of cerebral vessels are highly related to brain diseases, brain function, and brain development (Andreone et al. 2015; Sweeney et al. 2018). Therefore, visualizing, reconstructing, and analyzing the complete vascular structure down to the capillaries at the whole-brain scale is significant for understanding the brain. With the development of imaging technology, whole-brain optical microscopy imaging technology has been able to image the entire mouse brain at a single capillary resolution, providing an invaluable database for analyzing brain vasculature.

Vessel segmentation is a fundamental step of vessel reconstruction, and the accurately segmented vessel is a prerequisite for vascular analysis. Segmentation of high-resolution whole-brain vascular images remains a huge challenge. First, the amount of 3D image generated by whole-brain imaging at the capillary resolution is very large. For example, sub-micron imaging of a single mouse brain generates over 10 TB of data (Li et al. 2019). Because of individual differences, vascular analysis often requires a large number of samples. Duplication of samples imaging, segmentation, and analysis make the challenge of data size even more severe. How to rapidly segment massive amounts of vascular data has become a bottleneck for researchers. Secondly, vessels in different brain regions acquired by whole-brain high-resolution imaging vary widely in size, grayscale, and morphology. Traditional methods are difficult to identify all vascular features and segment them accurately. Therefore, an accurate and robust vessel segmentation algorithm is another bottleneck for whole-brain vessel segmentation.

Much work has been done on whole-brain vessel segmentation (Moccia et al. 2018; Jia and Zhuang 2021). Methods can be classified into three categories: threshold-based segmentation, filter-based or model-based traditional segmentation algorithms, and deep-learning-based segmentation. Wu et al. (2014), Xiong et al. (2017), and Zhang et al. (2019) used automatic or manual threshold segmentation methods to segment the vascular image collected by micro-optical sectioning tomography (MOST). Wälchli et al. (2021) used the OTSU algorithm to segment blood vessel imaging by µCT. They achieved vessel segmentation of specific brain regions or whole-brain vessel segmentation at low resolution. These threshold-based methods are very dependent on the image quality, which is difficult to achieve large-scale vessel segmentation, and the manual methods are less efficient. Ji et al. (2021) and Wu et al. (2022) used a series of filters to segment the vascular image generated by serial two-photon tomography (STP). Di Giovanna et al. (2018) used Markov random fields to segment the whole-brain vessels acquired by light-sheet fluorescence microscopy (LSFM). These traditional methods require designers to have the sufficient mathematical knowledge to design filters or models. For high-resolution whole-brain vascular images, it is difficult for a single model to extract vascular features from different regions of the whole brain. With the popularity of deep-learning methods in image processing, more deep-learning-based vessel segmentation methods have been proposed. Todorov et al. (2020) and Kirst et al. (2020) used a convolutional neural network (CNN) to implement the segmentation of whole-brain vessels acquired by LSFM. The benefit of deep-learning methods is that the vessel features can be automatically learned from images. However, most deep-learning-based methods do not consider computational efficiency and applicability to large-scale data when designing network models. Therefore, an efficient and accurate segmentation method for massive amounts of whole-brain capillary-resolution images is an urgent need in field studies.

To this end, we introduce HP-VSP, a high-performance deep-learning-based pipeline for whole-brain vessel segmentation. We designed a lightweight neural network model for multi-resolution vessel feature extraction and segmentation, which can achieve more accurate segmentation results with only 1% of the parameters of similar methods. We implemented the pipeline using parallel computing to improve the efficiency of vessel segmentation and the extendibility on various computing platforms. We used HP-VSP to segmentation of whole mouse brain vascular data acquired by HD-fMOST (Zhong et al. 2021). In addition, based on the pipeline, we segmented the vessels in the hippocampal region and reconstructed and analyzed the segmented vessels to demonstrate that our proposed pipeline can be used for applications of vascular analysis.

## 2 Materials and methods

### 2.1 Whole-brain vascular labeling and imaging

We used Tek-Cre::Ai47 transgenic mice to label the whole-brain vasculature. This mouse is a cross between a Tek-Cre mouse and an Ai47 mouse. In this transgenic mouse, the endothelial cells in the vascular wall were labeled with green fluorescent protein (GFP). We removed and embedded the mouse brains for imaging. Detailed sample preparation can be found in our previous works (Chen et al. 2022; Zhang et al. 2022). All animal experiments followed procedures approved by the Institutional Animal Ethics Committee of Huazhong University of Science and Technology.

We used HD-fMOST to image the whole mouse brain. During imaging, we used propidium iodide (PI) to real-time label cytoarchitecture. The system could simultaneously acquire vessel structures (green channel) and cytoarchitecture information (red channel) by two-channel imaging. The resolution of the vessel channel is 0.32 µm × 0.32 µm × 1 µm, and the resolution of the cytoarchitecture channel is 1 µm × 1 µm × 1 µm. The uncompressed data size of the two acquired channels is 19.8 TB.

Four 8-week-old mice were used in this article. One dataset was used to make the training dataset, and the remaining three datasets were used for experiments and analysis.

### 2.2 Whole-brain vessel segmentation pipeline

**Figure 1.**
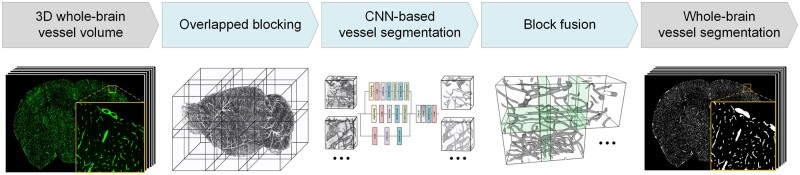
The overview of HP-VSP architecture. The pipeline consists of three steps: (1) whole-brain volume overlapped blocking; (2) vessel block segmentation based on 3D-CNN; (3) segmented block fusion

To achieve fast and accurate segmentation of whole-brain vessels, we propose a high-performance vessel segmentation pipeline based on deep learning called HP-VSP. The pipeline consists of three parts: whole-brain volume overlapped blocking, vessel block segmentation based on 3D-CNN, and segmented block fusion, as shown in Fig. 1.

1. Whole-brain volume overlapped blocking. High-resolution imaging of the whole brain produces hundreds of gigabytes (GB) or even TB of volume data. It is difficult for the computer to read and segment all data at once. Therefore, we used the divide-and-conquer strategy to handle the large-scale volume. We split the volume into equal-sized blocks (192 × 192 × 192 pixels) in 3D and recorded each block’s coordinate information. The two neighboring 3D blocks have a fixed overlapped area (32 pixels) to avoid inaccuracies at the chunk boundaries.
2. Vessel block segmentation based on 3D-CNN. After overlapped blocking, we segment each vessel block. Due to the complexity of the features of whole-brain vessels, we use neural networks to learn the vessel features and segment them. We designed a lightweight segmentation network with multi-resolution vessel feature extraction. This network can learn vessel features at different scales and perform segmentation with a small number of network parameters. See the following sub-section for details.
3. Segmented block fusion: This step restores the segmented vessel blocks to the original data space according to the coordinate information. Since the blocking operation truncates the vessels at the borders of blocks, some isolated vessel segments will be formed. The incomplete vessel structure will cause a loss of vessel features and make the segmentation results inaccurate. In addition, isolated background fluorescence noises in the image also affect the segmentation results. Therefore, in the fusion step, we use hole filling and isolated regions removal to fill possible cavities in very large vessels or tiny isolated noises. Finally, a segmented whole-brain vasculature is obtained and saved as 2D TIFF slices.

### 2.3 Multi-resolution feature extraction segmentation networks

High-resolution imaging of whole-brain vasculature could capture both large vessels of hundreds of microns in diameter and capillaries of a few microns in diameter. Vessel characteristics are different according to the diameter of the vessel. For example, because the labeling method labeled endothelial cells in the vascular wall, there are obvious cavities in the center of the large vessels. In contrast, the cavities in the center of the small vessels are not obvious. The fluorescence signal of small vessels is weaker compared to large vessels, and the curvatures and branches of small vessels are more complex than those of large vessels. To achieve accurate segmentation of all vessels simultaneously, we propose a lightweight segmentation network with multi-resolution feature extraction. The network can extract and fuse different scales of vessel features to accurately segment vessels with fewer network parameters. The efficient network structure makes it more suitable for segmenting large-scale data.

**Figure 2.**
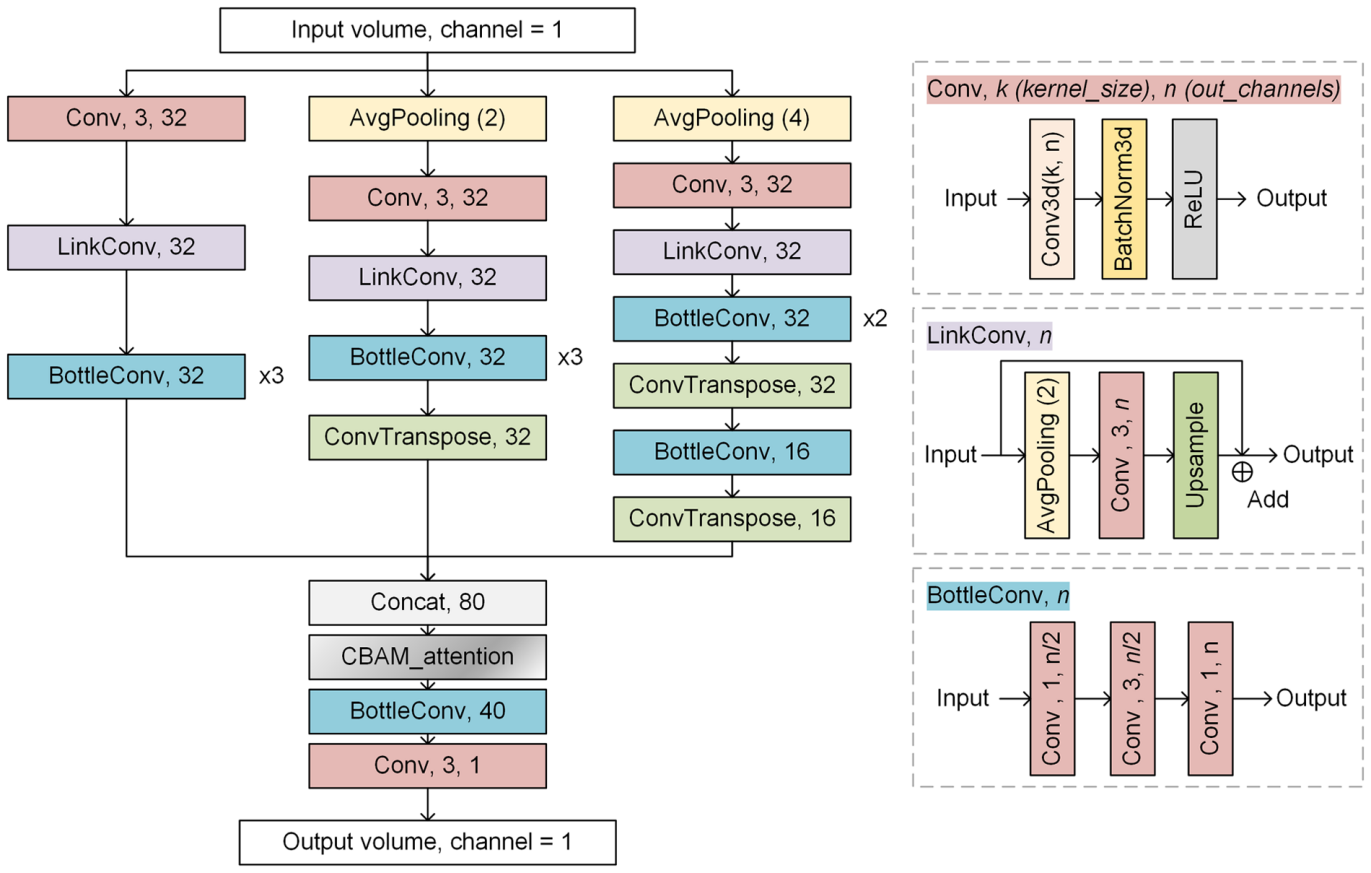
The architecture of the proposed multi-resolution feature extraction segmentation network

The schematic diagram of the network structure is shown in Fig. 2. We downsampled the original volume two times and four times with two avgpooling layouts, respectively. As the down-sampling rate increases, the cavities in the large vessels will become smaller, making the cavities less visible, but the detailed information about microvessels will be lost. Therefore, we extracted vessel features by convolution at three different resolutions: original resolution, 1/2 downsampling, and 1/4 downsampling. In the features extraction, we borrowed the idea of U-Net (Çiçek et al. 2016) and designed a LinkConv module to extract depth features by downsampling–convolution–upsampling combined with skip-linking. In addition, to reduce the number of network parameters, we used the idea of Bottleneck (He et al. 2016) to design a BottleConv module. BottleConv could reduce the number of network parameters and improve the feature extraction ability by deepening the network depth. After extracting the features, the 1/2 and 1/4 downsampled feature maps are deconvoluted to the original data resolution and concatenated. Then, a convolutional block attention module (CBAM) (Woo et al. 2018) is used to adjust feature weights for different channels and spatial locations of the feature maps. Finally, the number of channels of the feature maps is changed to one (final output) by two consecutive convolution layouts.

### 2.4 Post-processing to improve the segmentation effect

There will be some over-segmented and under-segmented regions in the segmentation results. We used morphological operations to post-process the segmentation results to improve the results’ accuracy.

**Figure 3.**
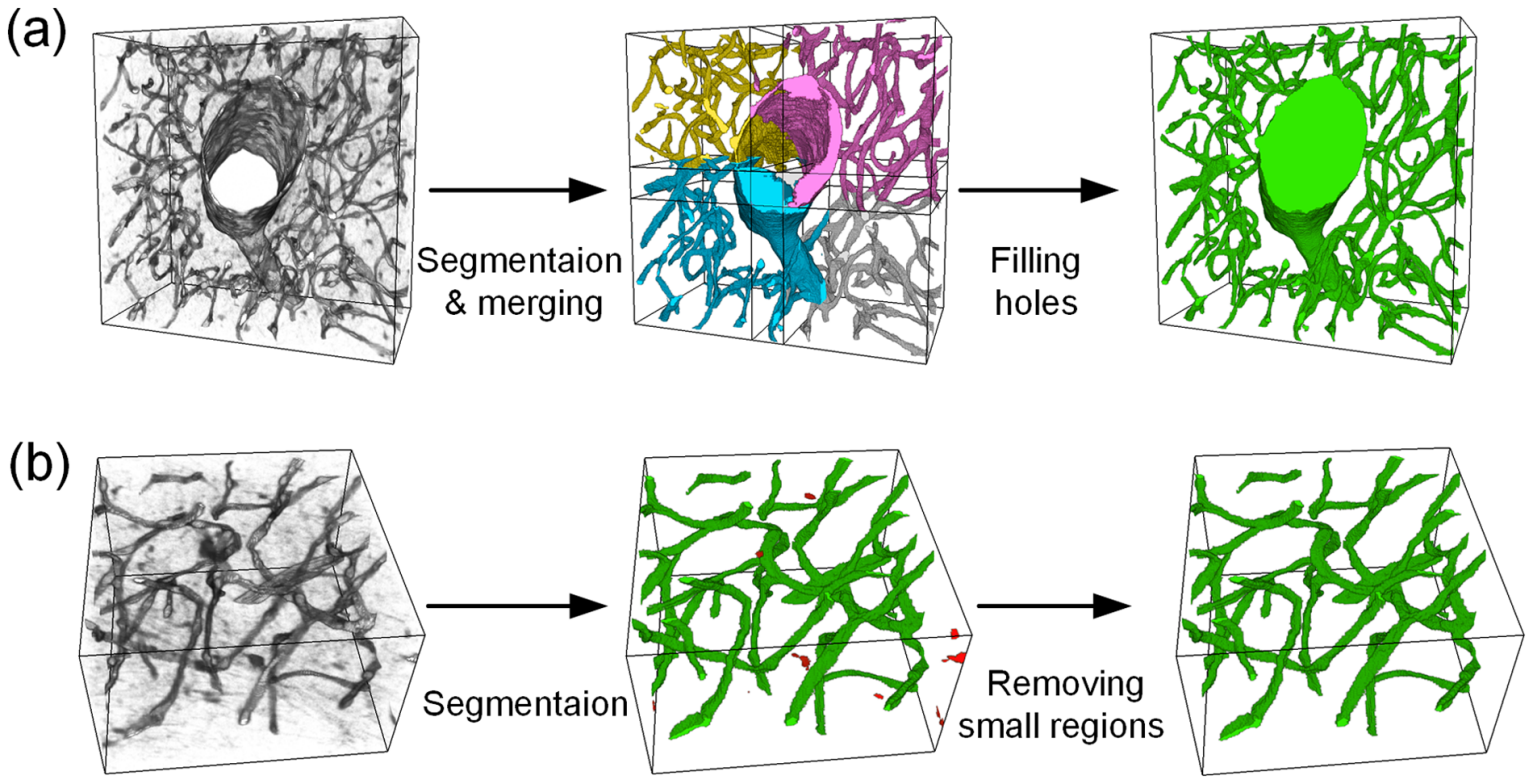
Post-processing operations. (a) Vessel lumens filling. (b) Tiny connectivity domains removal

Due to the great variety in size of brain vessels, the blocking process may cause a large vessel to be split into different blocks. The vessel features in these blocks are incomplete, making it difficult to accurately segment the vessels (identify and fill the vessel lumens). However, the network could accurately segment the vessel walls. After merging the blocks, a closed cavity area is formed in the center of the large vessel. We repeated six successive 2D hole-filling operations (x–y plane, x–z plane, and y–z plane, twice) on the merged 3D image. The filling operations could effectively fill the under-segmented area in the middle of the large vessels (Fig. 3a).

Some isolated fluorescence signals in the image background may result in isolated noise in the segmentation results. Since there are no isolated vascular segments in the brain, real vessels are connected together to form a network. According to this characteristic, we performed the connectivity domain analysis on the merged 3D image and removed the connectivity domains with the number of pixels below a threshold value (Fig. 3b). As a result, the isolated over-segmented regions were eliminated.

### 2.5 Parallel acceleration of large-scale volume segmentation

**Figure 4.**
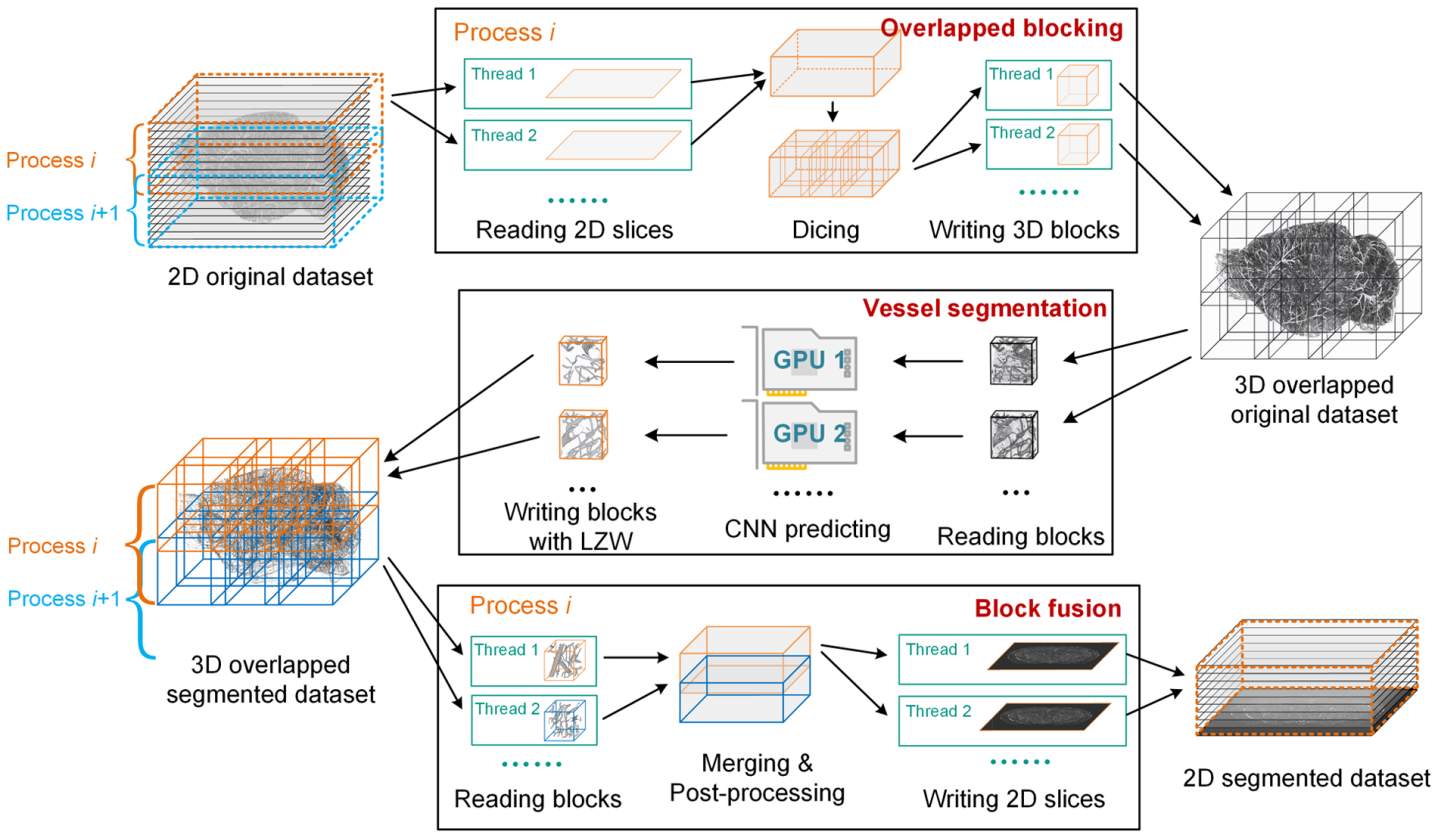
The acceleration of whole-brain segmentation pipeline using parallel computing

We accelerate the proposed vessel segmentation pipeline using parallel computing techniques (Fig. 4). This parallel acceleration improves the efficiency of terabyte-scale volume segmentation.

In the step of overlapped blocking, we divided the original volume into different overlapped sections in the z-direction. We used multiple processes to read the slices in the corresponding sections separately. In each process, multiple threads are used to read different slices simultaneously. After each process completes the reading, slices are merged into a section, and the section is divided into blocks according to the blocks’ coordinates. Then, the blocks are written out using multiple threads, and the offset coordinates of each block are recorded into a txt file.

In block segmentation, we used multiple GPUs to accelerate the neural network’s prediction. The blocks are read into memory, and one to three blocks (depending on the size of VRAM) form a batch fed into a GPU. After the segmentation, the segmented volumes are written out to the disk. LZW lossless compression is used to save the binarized images, which could reduce the amount of data size and improve the efficiency of file writing.

The block fusion step is the reverse of overlapped blocking. First, the segmented blocks are divided into different sections according to the z-direction index, and multiple processes are used to read the blocks in the corresponding sections separately. Each process reads all the blocks in the two contiguous sections at once. Once the reading is complete, the blocks are combined according to offset coordinates. The overlapping areas are combined using the ‘OR’ operation. In each process, the two merged sections are performed by post-processing. Finally, the merged section is written to the disk according to the z-direction and saved as continuous image slices. Since each process reads two adjacent sections, the writing process only saves all slices of the first section. The slices of the second section are saved by the adjacent process.

We use MPI, a common parallel computing framework, to implement parallelized processing, allowing our pipeline to be used on various computing platforms, such as common computers or high-performance clusters.

### 2.6 Training data preparation and implementation details

To prepare the experimental dataset for network training, we first resampled the resolution of vessel channel data to 1 µm × 1 µm × 1 µm and randomly cropped 179 blocks of size 160 × 160 × 160 pixels from them. One hundred and thirty-two of these blocks contain vessel signals, and the remaining 47 contain background signals only. The background blocks will not improve the segmentation effect (Supplementary Fig. S1). The randomly cropped blocks contain these background blocks, so we did not remove these blocks. We used the segmentation module in Amira to manually annotate these original blocks and generated binarized blocks as ground truths. One hundred and seventy-nine blocks were divided into training (70%), validation (15%), and testing (15%) datasets.

We implemented the pipeline in Python, where the segmentation network was implemented using Pytorch and the parallel computing using the mpi4py library and threading library. In the network training stage, the Adam algorithm was used for optimization, with an initial learning rate of 0.001, dropping to 98% of the previous value every 10 epochs. The BCE loss was used to train the neural network. Due to the small number of training datasets, the data were augmented with random cropping, random transposition, and random brightness changes.

We tested our segmentation pipeline on two different computing platforms, a desktop, and a high-performance cluster (HPC), to verify the efficiency of our approach. The computer platforms are configured as shown in Supplementary Table S1.

## 3 Results

### 3.1 Whole-brain vascular imaging

**Figure 5.**
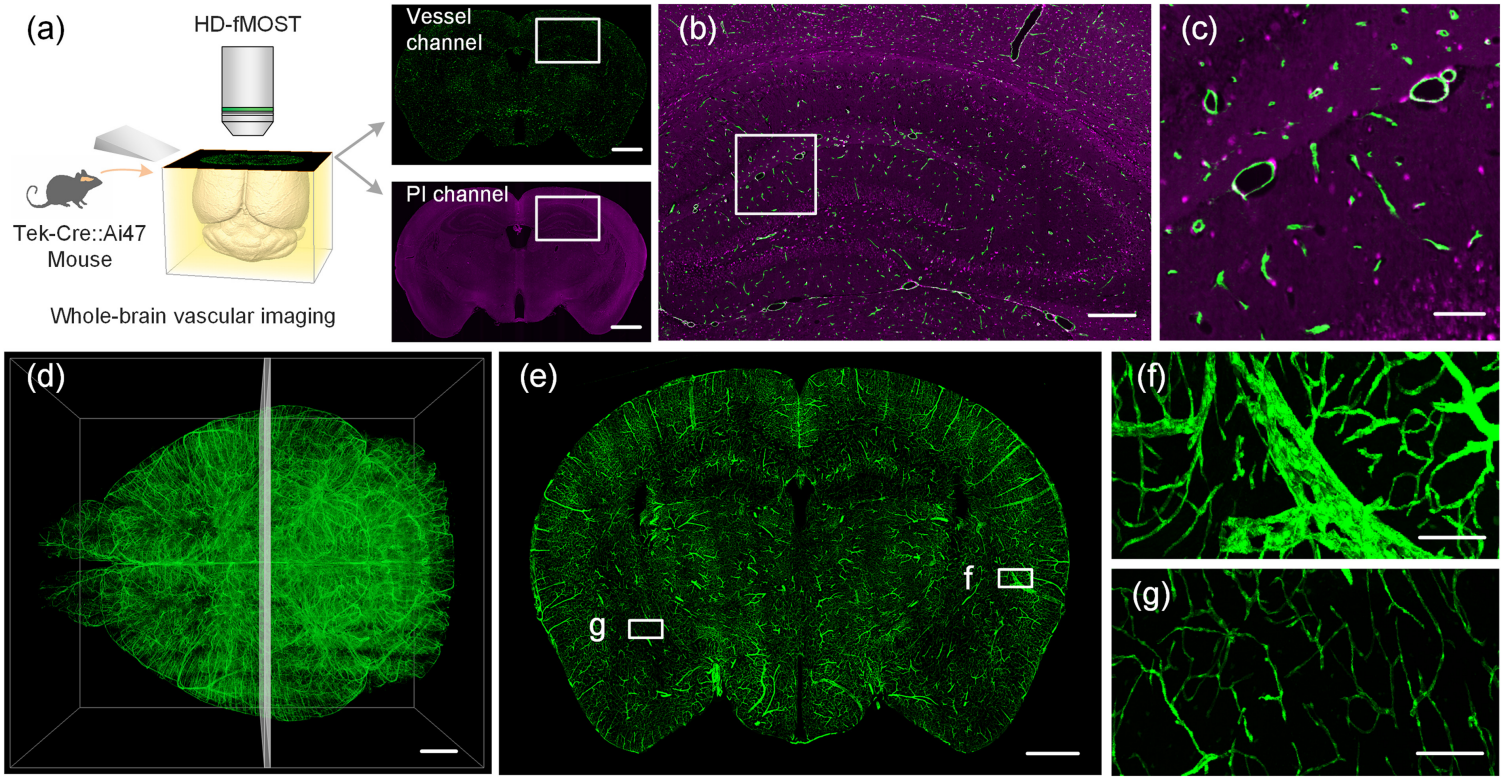
Two-channel whole-brain vascular imaging. (a) HD-fMOST to obtain a whole Tek-Cre::Ai47 mouse brain vasculature with cytoarchitecture. (b) Merged images indicated by the white box in (a). (c) An enlarged view of the area indicated by the white box in (b). (d) 3D visualization of the whole-brain vessel channel. (e) Coronal section of the whole-brain vasculature. Maximum intensity projections with 100 µm thickness. The selected coronal section is marked in (d). (f, g) Enlarged views of the areas indicated by the white box in (e). Scale bar, (a) 1000 µm, (b) 200 µm, (c) 50 µm, (d, e) 1000 µm, (f, g) 100 µm

We used HD-fMOST to obtain four whole-mouse-brain vascular datasets. Since HD-fMOST is two-channel imaging (Fig. 5a), the acquired dataset contains vessel information (Vessel channel) and cytoarchitecture information (PI channel). The vessel channel is GFP-labeled endothelial cells in the vessel wall, which contains all vascular structure information. PI channel marks the nucleus of all cells. Cells in different brain regions show density and structural differences in the images. So, PI channels can obtain cytoarchitecture information. As shown in Fig. 5b, the typical structure of the hippocampus can be easily identified. The Vessel channel and PI channel are imaged simultaneously (Fig. 5c) so that the whole-brain vasculature can be localized according to the cytoarchitecture of the PI channel, enabling the analysis of vessels in different brain regions.

Due to the entire brain imaging at high resolution allows it to obtain a complete and detailed whole cerebral vascular network from large arteries and veins to small capillaries (Fig. 5d and e). There is a significant difference in vessels’ morphological structure and fluorescence signal intensity. As shown in Fig. 5f and g, large vessels are bright but have cavities in the middle of the vessels, while capillaries are the opposite. Therefore, vessel segmentation methods are needed to accurately segment all vessels throughout the brain.

### 3.2 Performance evaluation of the segmentation network

#### 3.2.1 Segmentation results

**Figure 6.**
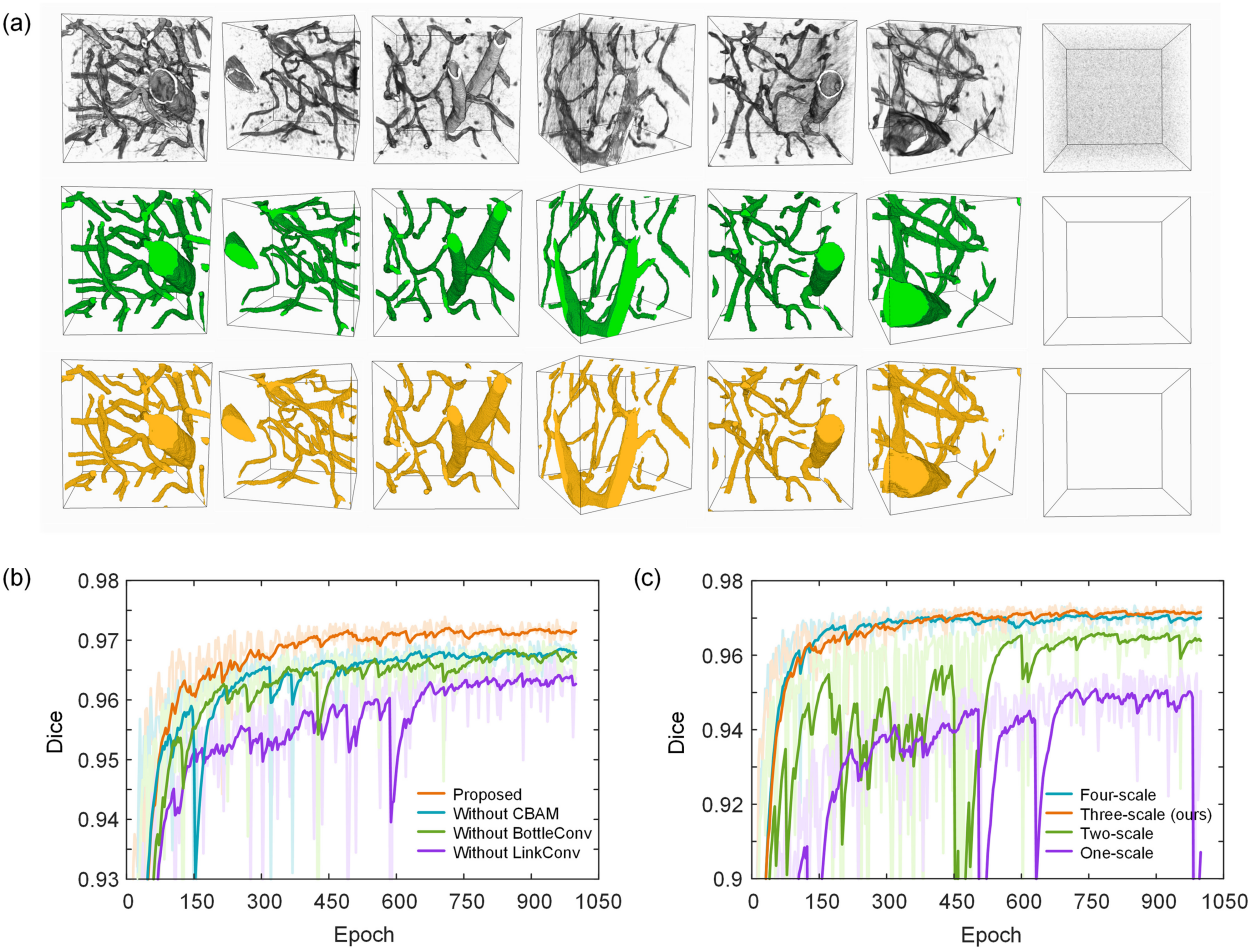
Segmentation results and ablation studies. (a) Examples of segmented vessels. The first row is the original vessel volume, the second row is the ground truths, and the third row is the predicted results of the network. The last column shows a block containing only background signals. The size of the blocks is 160 × 160 × 160 pixels. (b) Effectiveness of different modules. (c) Effectiveness of different-scale networks. Ablation experiments were performed on the validation set

We validated the performance of the proposed segmentation network on the test set. The segmentation results are shown in Fig. 6a. The original vessel volumes, the ground truths, and the predicted results are shown separately. It can be seen that our proposed multi-resolution feature extraction segmentation network could effectively segment all vessels. The predicted results are very close to the ground truths. For thick vessels, the model is able to identify and fill the cavities in the center of the vessels. For thin vessels, the weak fluorescent signal can be identified and accurately segmented to ensure the connectivity of the vessels. In addition, fluorescent noise in the background and nonvascular regions could also be identified effectively.

We quantitatively evaluated the segmentation results using the evaluation metrics and compared them with four other methods. The evaluation metrics are described in Supplementary Methods. The comparative visualization results are shown in Supplementary Fig. S2. The Quantitative results are shown in Table 1. For the pixel overlap-based evaluation metrics, our approach has a balanced in *Precision* and *Recall*, both of which achieve a high score, so the *Dice* (*F1-score*) value exceeds 0.97, and the *Jaccard* is close to 0.95. Both metrics achieve the highest compared to similar methods. On the other hand, our approach also performs well in terms of morphological similarity-based metrics. The *HD* (Hausdorff distance) is the highest, and the *clDice* is just a bit lower than MPL, indicating that our method effectively preserves the topology of the vessels. The segmentation effect could be further improved with the addition of post-processing operations. Moreover, our network has fewer parameters and fewer multiply-accumulate operations (MACs) compared to similar models.

**Table 1.** Quantitative comparison of segmentation performances^a^

	*Precision*	*Recall*	*Dice*	*Jaccard*	*clDice*	*HD*	Parameters^b^	MACs^b^
FCNs (Long et al. 2015)	0.981	0.953	0.966	0.935	0.982	31.432	55.92M	861G
3D U-Net (Çiçek et al. 2016)	**0.984**	0.948	0.965	0.934	0.977	28.887	16.42M	2128G
V-Net (Milletari et al. 2016)	0.977	0.953	0.964	0.931	0.966	40.175	45.60M	751G
MPL (Li et al. 2022)	0.970	0.965	0.967	0.936	0.990	28.138	82.92M	2721G
Our network	0.980	0.967	0.973	0.949	0.988	26.839	**0.15M**	**87G**
Our network and post-process	0.981	**0.968**	**0.974**	**0.950**	**0.990**	**17.120**		

a The detailed numerical data can be found in Supplementary Tables S2–S7.

b The parameters and MACs consumption is calculated using the thop library.

Note: Bold indicates the best performance.

In general, our approach achieved even better segmentation results with fewer network parameters and lower computational complexity, indicating that our method is more efficient.

#### 3.2.2 Ablation studies

Several designed modules are used in the proposed network to improve the segmentation effect. A multi-scale structure is also used to enhance the segmentation of vessels at different scales. We performed two ablation experiments to verify the effects of these structures on segmentation results.

First, we verified the effects of the LinkConv, BottleConv, and CBAM modules. We retrained the network after removing these modules (LinkConv and BottleConv were replaced by Conv module). The segmentation effect decreases significantly after removing these modules, especially LinkConv (Fig. 6b). LinkConv could extract and fuse two different-sized feature layers, allowing the network to learn richer features. The results show that each module is helpful for the improvement of the segmentation effect. Then, we verified the effect of the multi-scale (resolution) structure. We compared the performances of one-scale (1×), two-scale (1× + 2×), three-scale (our proposed), and four-scale (1× + 2× + 4× + 8×) networks, respectively. It can be seen that the performance of the multi-scale network is significantly better than that of a single-scale network, and the performance becomes better as the number of scales rises. However, there is almost no performance difference between the four-scale and three-scale networks (Fig. 6c). We choose the three-scale network. The performance and complexity of the network could be well balanced.

### 3.3 Whole-brain vessel segmentation and high-performance acceleration

**Figure 7.**
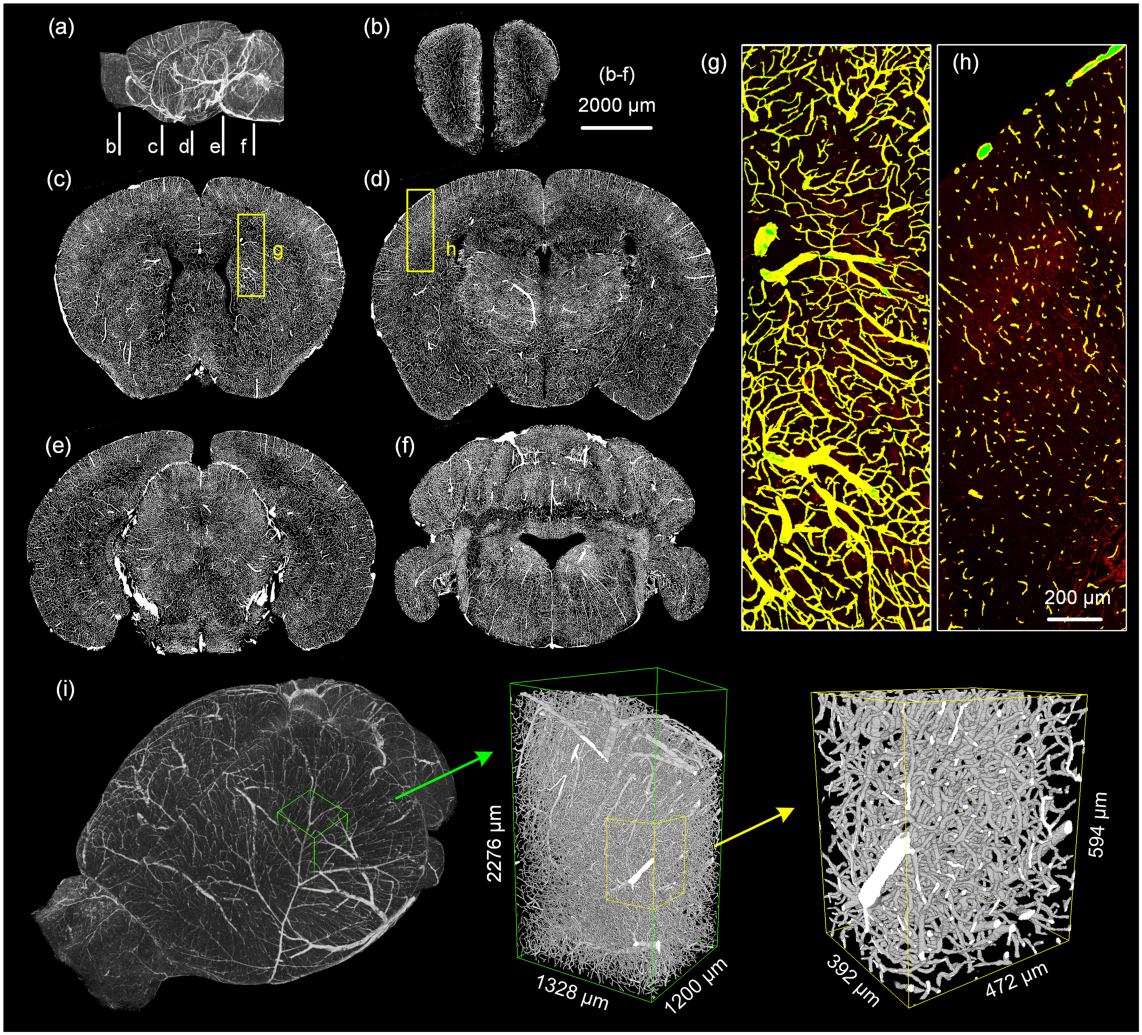
Whole-brain vessel segmentation results. (a) Sagittal view of segmentation results, b-f indicates the location of five coronal sections. (b)–(f) Maximum intensity projections of the coronal sections. The projection thicknesses are 80 µm. (g), (h) Two enlarged views in the yellow boxes in c, d. The thicknesses are 80 µm (g) and 2 µm (h), respectively. Segmentation results (green) are shown overlaid with the original data (red). Overlapping vessels are shown in yellow. (i) 3D visualization of whole-brain vessel segmentation results

We segmented the entire brain vessels using HP-VSP. The resolution of whole-brain vessel volume was downsampled to 2 µm × 2 µm × 2 µm. We select five different coronal sections from the segmented results for visualization (Fig. 7a–f). And two areas overplayed in the original images are selected from the sections (Fig. 7g and h). We also visualize the segmentation results in 3D and select two different-sized 3D volumes from the whole brain for display (Fig. 7i). It can be seen that the vessels in different regions can be accurately identified and segmented, and the segmented vessels are continuous in 3D. The noise in the background region can also be effectively removed. The results demonstrate that our proposed method can effectively segment whole-brain vessels.

To verify the segmentation efficiency of HP-VSP and its applicability to different platforms, we segmented the whole-brain data on two different computing platforms and counted the time consumption. The results are shown in Supplementary Table S8. It took 21.6 h to segment a whole-brain vessel dataset with 2 µm × 2 µm × 2 µm resolution on a desktop. In contrast, in an HPC with 11 nodes, it took just 2.7 h to complete a whole-brain vessel segmentation. Each step in the pipeline can be processed more efficiently through parallelism. The segmentation efficiency on HPC is significantly improved compared to the desktop. The results show that our proposed pipeline is highly extensible. It is able to achieve fast segmentation of big data by utilizing efficient computational resources.

### 3.4 Applications on the hippocampal vessel analysis

To verify that our segmentation method can be used for vessel reconstruction and analysis, we used the hippocampus as an example to segment, reconstruct, and analyze the vasculature in this brain region. The hippocampus plays an essential role in learning and memory, and quantitative characterization of the vasculature in this region could be helpful in the study of Alzheimer’s disease (Kisler et al. 2017; Zhang et al. 2019).

**Figure 8.**
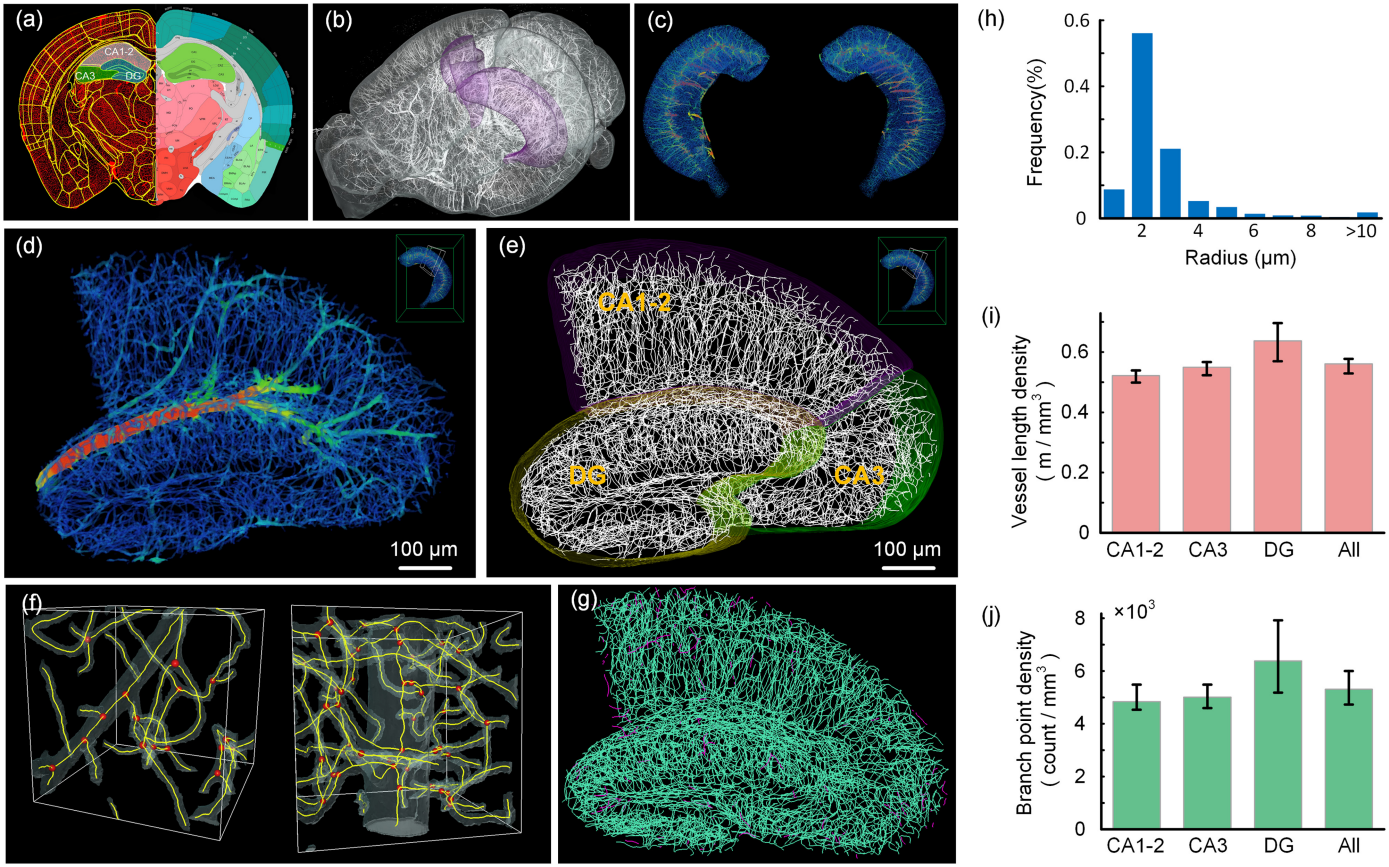
Results of vessel segmentation, reconstruction, and analysis in the hippocampus. (a) A coronal section from the registered whole-brain vessels. The right is the Allen CCFv3. The left half is the yellow lines of the brain region overlaid on the segmented images, and CA1-2, CA3, and DG are highlighted in different colors. (b) 3D visualization of segmentation results and locations of the hippocampus. (c) Segmentation of vessels in the hippocampus. The vessels are color-coded by diameters for clarity. (d) 3D visualization of a cross-section from the segmented vessels. (e) 3D visualization of the vessel skeleton superimposed on CA1-2, CA3, and DG borderlines. The centerlines are converted to SWC format for better visualization. (f) Two details of vessel centerlines and bifurcation points. The segmented vessels are shown as surface meshes. (g) Vessel connectivity domain analysis. The green centerlines all belong to one connectivity domain. The purple vessel centerlines belong to other connectivity domains. (h) The radius distribution of vessels in the hippocampal region. (i) Vessel length density across CA1-2, CA3, and DG. (j) Branch point density across CA1-2, CA3, and DG. Error bars in (i, j) represent the greatest and lowest values

We segmented and analyzed the vessels of the hippocampus in three 8-week-old mice. First, the whole-brain vascular dataset with 1 µm × 1 µm × 1 µm resolution was registered into the Allen mouse brain atlas. The hippocampal brain region was extracted based on the atlas and divided into three sub-regions CA1-2, CA3, and DG (Fig. 8a and b). Then, the vessels in these three sub-regions were segmented separately using HP-VSP. After segmentation, the centerlines and radii of vessels were extracted and calculated (Supplementary Methods and Supplementary Fig. S3), respectively (Fig. 8c–e). We show enlarged views of the centerlines and bifurcation points (Fig. 8f). We also did connectivity domain analysis on the centerlines in a section of the hippocampus and distinguished different connectivity domains with different colors (Fig. 8g). It can be seen that almost all of the vessels are connected except at the borders. These results confirm that our segmentation method could maintain the topology and connectivity of the vessels.

Based on the reconstructed skeletons, we first analyzed vessel size distribution. We counted the proportion of vessels of different sizes according to their radius (Fig. 8h). The majority of vessels are capillaries with radii of 2–3 µm. We also analyzed the vessel length density and bifurcation point density (Fig. 8i and j). The vascular length density in the hippocampus is 560 mm/mm^3^, and the bifurcation density is 5310 count/mm^3^. The detailed numerical data can be found in Supplementary Tables S9–S11. We compared the results with those of similar studies (Table 2). The results show that our segmentation method can be used for vascular analysis.

**Table 2.** Comparison of hippocampal vessel length densities

	Ji et al. (2021)	Kirst et al. (2020)	Todorov et al. (2020)	Xiong et al. (2017)	Ours
Labeling	FITC-BSA	CD31, acta2, podocalyxin	WGA and EB dye	Nissl	Tek-Cre::Ai47
Imaging	STP	LSFM	LSFM	MOST	HD-fMOST
Segmentation	Filters	Filters + CNN	CNN	Manual	CNN
Vessel length density (m/mm^3^)	0.71^a^	0.30^a^	0.45^a^	0.69^a^	0.56

a Reported by Ji et al. (2021).

## 4 Discussion

The proposed whole-brain vessel segmentation pipeline has two characteristics: the multi-resolution feature extraction segmentation network and the parallel computing-based acceleration of the pipeline. Our segmentation model aims to solve the problem of significant variation in whole-brain vessels. In our application, the characteristics of large and small vessels are inconsistent. Segmenting large vessels requires the network to learn semantic-level information (fill large hollows roughly). While segmenting small vessels requires the network to learn pixel-level information (precise identification of vascular boundaries and weak signals). Similar models like U-Net and V-Net adopt a feature multi-scale structure. The features at different scales are extracted by downsampling feature layers, while the features of different-sized vessels are extracted together. These features are fused together and may cause some confusion to the network during training. On the other hand, our proposed model adopts an image multi-scale by downsampling the original image. The network separates large and small vessels in different network branches (different resolution images) for feature learning, which could avoid interference between different-sized vessels during feature learning. The designed network is more specific than the general semantic segmentation network. To solve the problem of large-scale data, we adopt the strategy of divide-and-conquer, which splits the volume into small blocks, segments the blocks separately, and merges the segmented blocks. And the parallel computing approach is used to optimize the workflow, which allows the proposed pipeline to improve efficiency by leveraging high-performance clusters.

Although our method achieves good results, it still has some limitations. The main problem is the big hollow vessels. We trained the network to segment the vessel lumens directly as foreground. However, due to the limited receptive field of the network and small training samples of large vessels, it is challenging to segment the vessel lumens accurately for extremely large vessels. For vessels with radii below 20 µm, our network is able to identify the vessel lumens and fill them accurately (Supplementary Fig. S4). For larger vessels, our method could only segment the vessel wall, and the lumens are difficult to segment directly. These lumens will impact skeletonization, especially when there are holes in the vessel wall. If the vessel lumens are closed areas or the holes in the vessel walls are not very large, the post-processing operations and Gaussian smoothing operation could fill these hollows to obtain the correct skeletons (Supplementary Fig. S5). However, if these holes are large, automatic methods make filling them difficult. Further manual corrections are required, but fortunately, the percentage of these large vessels is not very high.

In the last of the results section, we show our segmentation pipeline used in the application of vascular analysis. The statistical results of vascular analysis depend on various factors, including sample preparation, labeling method, imaging quality, segmentation accuracy, skeletonization, and brain registration. As each step may generate some errors, the statistical results of the final analysis vary widely. For example, fluorescent dye perfusion labeling methods have the problem of incomplete filling of the vessel lumen compared with transgenic labeling (Bennett and Kim 2022), which can lead to the loss of vessels in the data after imaging. As another example, vessels on the brain’s surface may be crushed or damaged during sample preparation, resulting in vessel deformation. This makes the segmentation and reconstruction of the blood vessels on the brain’s surface less accurate. It is precise because so many factors affect the final results, vascular analysis requires many samples to repeat the experiment. Segmentation, reconstruction, and analysis of large amounts of datasets to produce statistically significant results. The large-scale data processing tasks could better highlight the advantages of our proposed high-performance vessel segmentation pipeline.

## 5 Conclusion

We propose HP-VSP, a high-performance vessel segmentation pipeline based on deep learning. The multi-resolution feature extraction segmentation network in the pipeline could accurately segment whole-brain vessels with only a small number of parameters and low computational complexity. The pipeline uses parallel computing to improve the efficiency of segmentation and the scalability of various computing platforms. We used the pipeline for accuracy and efficient segmentation and analysis of whole-brain vessels. We believe the proposed pipeline will be a powerful tool for vascular analysis, the construction of cerebrovascular atlases, and vascular disease-related research.

## Supplementary Material

btad145_Supplementary_Data
