# Supplementary material for: A high-performance deep-learning-based pipeline for whole-brain vasculature segmentation at the capillary resolution
Source: Bioinformatics. 2023 Mar 22;39(4):btad145. doi: 10.1093/bioinformatics/btad145 (PMC10068744; doi:10.1093/bioinformatics/btad145)
Supplement: btad145_Supplementary_Data [file btad145_supplementary_data.pdf]

Supplementary Materials

A high-performance deep-learning-based pipeline for whole-brain vasculature segmentation at the capillary resolution

Yuxin Li[1,*], Xuhua Liu[1], Xueyan Jia[3], Tao Jiang[3], Jianghao Wu[1], Qianlong Zhang[1], Junhuai Li[1], Xiangning Li[2,3], Anan Li[2,3,*]

[1]*Shaanxi Key Laboratory of Network Computing and Security Technology, School of Computer Science and Engineering, Xi'an University of Technology, Xi'an, 710048, China*
[2]*Britton Chance Center for Biomedical Photonics, Wuhan National Laboratory for Optoelectronics, MoE Key Laboratory for Biomedical Photonics, Huazhong University of Science and Technology, Wuhan, 430074, China*
[3]*HUST-Suzhou Institute for Brainsmatics, Suzhou, 215123, China*
[*]*Corresponding author: liyuxin@xaut.edu.cn & aali@hust.edu.cn*

Supplementary Methods

(1) Evaluation metrics

We used the trained neural network to predict the blocks in the test set. The predictions were binarized according to a threshold of 0.5, and compared the binarized results to the ground truths. Commonly used segmentation evaluation metrics are employed for quantitative analysis of the results, including *Precision, Recall, Dice, Jaccard, clDice, and Hausdorff Distance*. The first four are pixel overlap-based metrics, and the last two are morphological similarity-based metrics. The evaluation metrics are described below:

TP is the number of vessel pixels correctly detected.

FP is the number of vessel pixels incorrectly detected.

TN is the number of non-vessel pixels correctly detected.

FN is the number of non-vessel pixels incorrectly detected.

Then,

Precision is defined as $Prec = TP / (TP + FP)$.

Recall is defined as $Rec = TP / (TP + FN)$.

$F1$-*score* or *Dice* is the harmonic mean of *Precision* and *Recall*, and it is defined as $F1 = 2TP / (2TP + FP + FN)$.

Jaccard or *IoU is* defined as $Jaccard = TP / (TP + FP + FN)$.

the *Hausdorff* distance between A and B is defined as $H(A, B) = \max(h(A, B), h(B, A))$, where $h(A, B)$ is the *Hausdorff* distance from set A to set B, defined as $h(A, B) = \max_{a \in A}(\min_{b \in B}(d(a, b)))$, where a and b are points of sets A and B respectively, and $d(a, b)$ is any metric between a and b.

clDice measures the topological similarity of the segmentation results and the ground truths. The introduction of *clDice* can be found in [1].

(2) Brain registration and vessel analysis

We have performed registration, segmentation, skeletonization, and quantitative analysis operations on mouse hippocampal brain regions to demonstrate that the proposed pipeline can be applied for whole-brain vessel reconstruction and analysis. The whole-brain datasets are registered into the Allen Mouse Brain Common Coordinate Framework (CCFv3) by using BrainsMapi [2]. The skeletonize_3d module in the scikit-image library is used to skeletonize the segmented vessels. Before skeletonizing, the segmented vessels are smoothed by three successive 3D Gaussian blurs (sigma: 1). The smoothed vessels are then segmented using a fixed threshold (gray value: 60). These operations could reduce the negative impact of coarse vessel surfaces on skeletonization (**Fig. S3**). A distance transformation is performed on the segmented vessels, and then the vessel radius information is obtained by multiplying the skeletonization with the distance transformation matrix (element-wise multiplication). The bifurcation points and vessel length statistics are done using the Fiji analysis skeleton module [3].

References

[1] S. Shit, et al. (2021) clDice-a novel topology-preserving loss function for tubular structure segmentation. In, *Proceedings of the IEEE conference on computer vision and pattern recognition.* 16560–16569.

[2] Ni, H., et al. (2020) A Robust Image Registration Interface for Large Volume Brain Atlas. Scientific reports,10,2139.

[3] Schindelin, J., *et al.* (2012) Fiji: an open-source platform for biological-image analysis. *Nature methods*,9,676-682.

Supplementary Figures

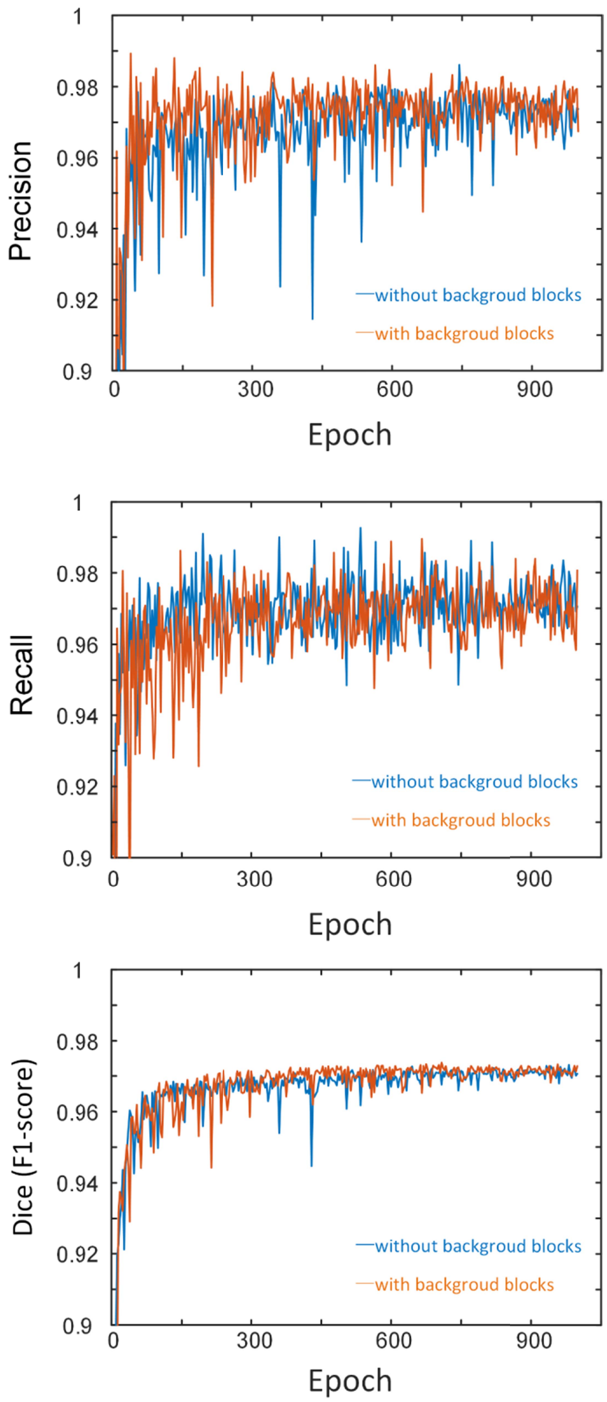

Fig. S1. Performance comparison on the validation set with and without background blocks.

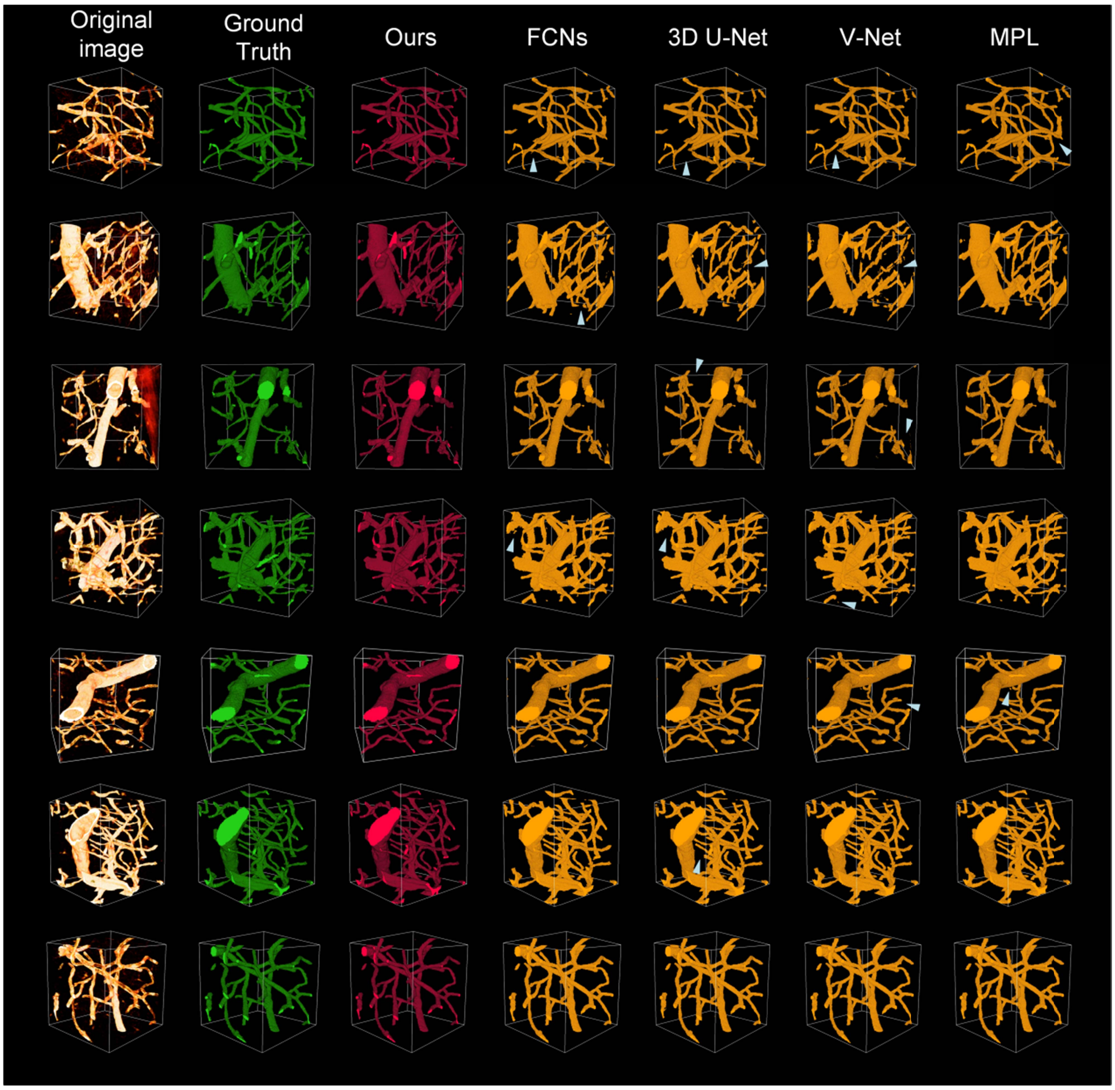

Fig. S2. Comparative segmentation results using different methods on test data. Some false segmentations are highlighted with arrows. Volume size: 160 × 160 × 160 µm.

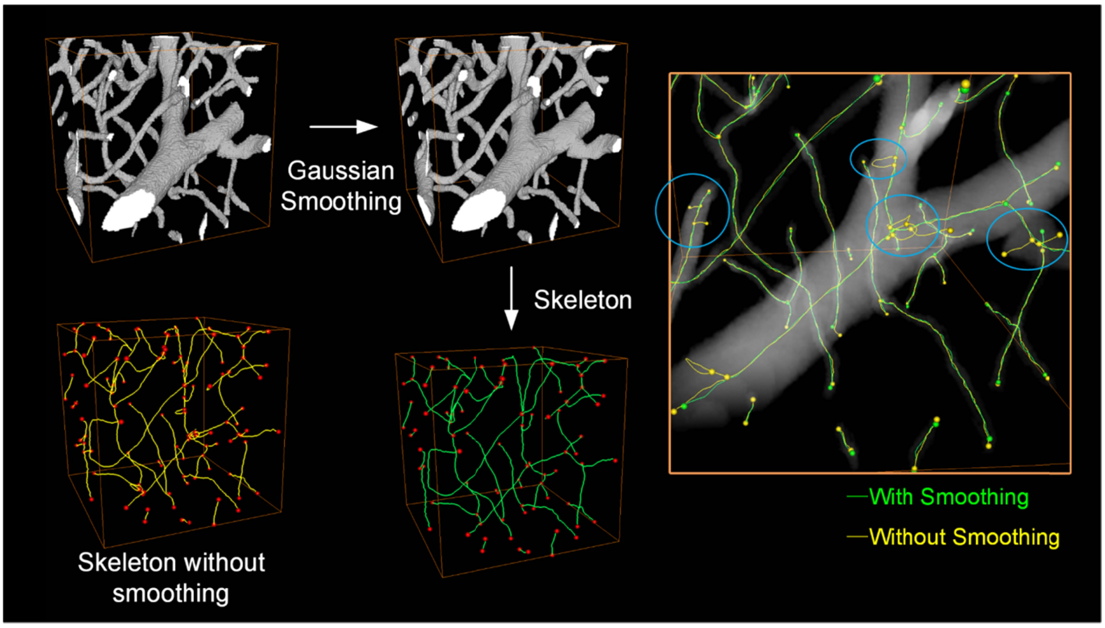

Fig. S3. Gaussian smoothing and vessel skeletonization.

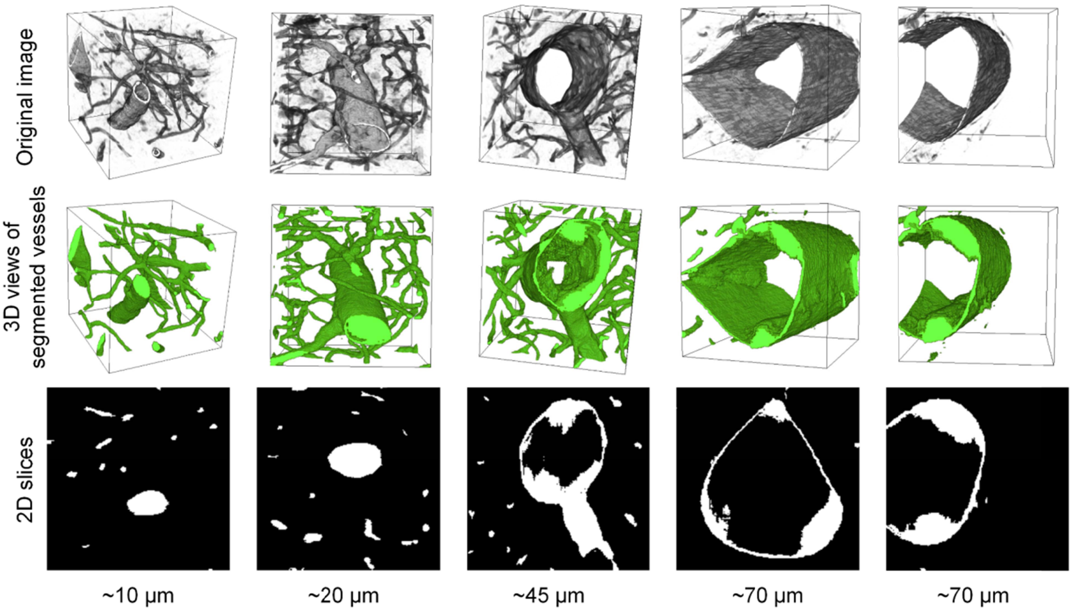

Fig. S4. The results of large vessel segmentation. The values in the bottom row are the vessel radius. The last two columns are the same vessel, one in the middle of the block and one at the boundary. Volume size: 160 × 160 × 160 μm.

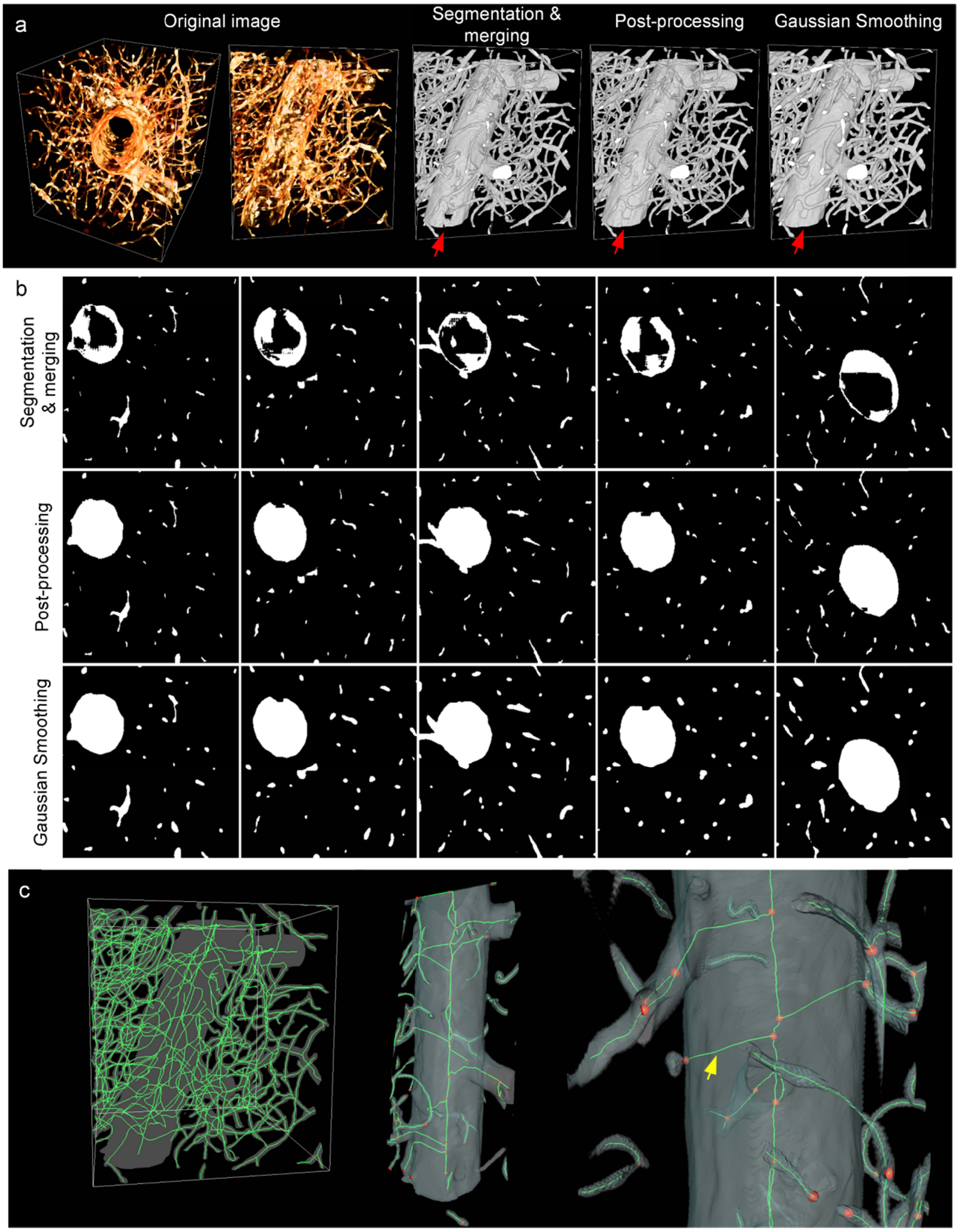

Fig. S5. Segmentation, post-processing, and skeletonization of large vessels. (a) 3D visualization of post-processing and smoothing results. The red arrow points to the holes in the vessel wall. (b) 2D visualization of post-processing and smoothing results. (c) Skeletonization results. The yellow arrow points to the wrong branch. Data size: ~ 300 × 330 × 320 μm.

Supplementary Tables

Table S1. Detail information of the computers' configuration.

	Desktop	Cluster (10 nodes +1 GPU server)
CPU	Intel Core i9-9900K	Intel Xeon Gold 6248 × 2
RAM	128 GB	256 GB
GPU	RTX 3090 24 GB	Tesla V100 32 GB × 8
Storage	12 TB HDD	Lustre file system 17 PB
OS	Windows 10	Linux Centos 7

Table S2. The performance of the proposed network on test data.

Cases	Precision	Recall	Dice	clDice	Hausdorff	Jaccard
block_3044_4250_9739_ch1.tif	0.990924	0.963049	0.976788	0.987389	39.31921	0.954629
block_3782_3113_3259_ch1.tif	0.974211	0.99683	0.985391	0.980402	35.36948	0.971202
block_3806_5055_4879_ch1.tif	0.995989	0.917206	0.954975	0.990386	18.54724	0.91383
block_4399_3821_4339_ch1.tif	0.964341	0.9936	0.978752	0.980516	27.60435	0.958388
block_4428_5129_3259_ch1.tif	0.987596	0.991679	0.989633	0.993861	26.07681	0.979479
block_4603_5384_4879_ch1.tif	0.924912	0.979171	0.951269	0.985374	24.73863	0.907066
block_4787_3606_4079_ch1.tif	0.986386	0.985421	0.985903	0.982108	40.26164	0.972198
block_5041_3791_8479_ch1.tif	0.984627	0.901118	0.941023	0.981417	40.16217	0.888615
block_5089_4849_5239_ch1.tif	0.957208	0.991019	0.97382	0.990772	11.83216	0.948976
block_5207_4811_3259_ch1.tif	0.985769	0.969898	0.977769	0.997331	46.76537	0.956505
block_5582_3923_3799_ch1.tif	0.996329	0.983341	0.989793	0.997904	45.04442	0.979792
block_5981_4531_2539_ch1.tif	0.993748	0.904429	0.946987	0.988556	22.04541	0.899312
block_5997_5939_6319_ch1.tif	0.99793	0.977036	0.987373	0.992273	2.236068	0.97506
block_6009_5240_3539_ch1.tif	0.928663	0.996	0.961153	0.987952	24.37212	0.925212
block_6083_2958_3619_ch1.tif	0.976165	0.984843	0.980485	0.983995	20.80865	0.961717
block_6084_2665_6679_ch1.tif	0.98019	0.976612	0.978398	0.984859	35.69314	0.957709
block_6463_4389_4259_ch1.tif	0.987017	0.991175	0.989091	0.991653	29.79933	0.978418
block_6619_4151_4619_ch1.tif	0.996799	0.9104	0.951643	0.987872	7	0.907747
block_7547_3960_6599_ch1.tif	0.989844	0.959335	0.974351	0.991478	49.09175	0.949984
block_7708_3900_5159_ch1.tif	0.983338	0.976138	0.979725	0.98396	33.97058	0.960256
block_7751_5462_5519_ch1.tif	0.991162	0.94895	0.969597	0.994363	3.316625	0.940988
block_7882_5182_5519_ch1.tif	0.994253	0.986718	0.990471	0.99219	6.403124	0.981122
**Average**	**0.980336**	**0.967453**	**0.973381**	**0.988482**	**26.83901**	**0.948555**

Table S3. The performance of the proposed network+post-processing on test data.

Cases	Precision	Recall	Dice	clDice	Hausdorff	Jaccard
block_3044_4250_9739_ch1.tif	0.990978	0.96375	0.977175	0.987983	10.04988	0.955368
block_3782_3113_3259_ch1.tif	0.975111	0.997032	0.98595	0.982168	10.19804	0.972289
block_3806_5055_4879_ch1.tif	0.996038	0.917735	0.955284	0.99059	9.69536	0.914397
block_4399_3821_4339_ch1.tif	0.96524	0.99411	0.979462	0.98373	27.60435	0.95975
block_4428_5129_3259_ch1.tif	0.987527	0.992172	0.989844	0.993634	5.744563	0.979893
block_4603_5384_4879_ch1.tif	0.925269	0.979667	0.951691	0.987653	32.18695	0.907835
block_4787_3606_4079_ch1.tif	0.986981	0.985965	0.986473	0.984806	45.70558	0.973307
block_5041_3791_8479_ch1.tif	0.985338	0.901849	0.941746	0.98361	13.60147	0.889906
block_5089_4849_5239_ch1.tif	0.956962	0.99133	0.973843	0.995441	4	0.94902
block_5207_4811_3259_ch1.tif	0.985641	0.970639	0.978082	0.998052	2.44949	0.957105
block_5582_3923_3799_ch1.tif	0.996516	0.983697	0.990065	0.999359	1.732051	0.980325
block_5981_4531_2539_ch1.tif	0.994183	0.904707	0.947337	0.990159	5.744563	0.899943
block_5997_5939_6319_ch1.tif	0.99788	0.978127	0.987905	0.992273	2.236068	0.976099
block_6009_5240_3539_ch1.tif	0.928515	0.996434	0.961276	0.989813	5.09902	0.925439
block_6083_2958_3619_ch1.tif	0.977509	0.986659	0.982063	0.988779	21.0238	0.964758
block_6084_2665_6679_ch1.tif	0.979906	0.978885	0.979396	0.980937	35.69314	0.959623
block_6463_4389_4259_ch1.tif	0.987213	0.992042	0.989622	0.990625	65.87109	0.979457
block_6619_4151_4619_ch1.tif	0.996957	0.911527	0.95233	0.989792	11.7047	0.908998
block_7547_3960_6599_ch1.tif	0.990116	0.960091	0.974872	0.994545	6.403124	0.950976
block_7708_3900_5159_ch1.tif	0.983622	0.976787	0.980193	0.987543	6.480741	0.961155
block_7751_5462_5519_ch1.tif	0.990968	0.949407	0.969743	0.993448	7.483315	0.941262
block_7882_5182_5519_ch1.tif	0.993927	0.98665	0.990275	0.989239	45.93474	0.980738
**Average**	**0.980563**	**0.968148**	**0.973847**	**0.989736**	**17.12009**	**0.949438**

Table S4. The performance of 3D U-Net on test data.

Cases	Precision	Recall	Dice	clDice	Hausdorff	Jaccard
block_3044_4250_9739_ch1.tif	0.997958	0.908973	0.951389	0.972785	38.74274	0.907286
block_3782_3113_3259_ch1.tif	0.979849	0.976555	0.9782	0.922229	35.36948	0.957329
block_3806_5055_4879_ch1.tif	0.998779	0.901103	0.94743	0.992061	48.38388	0.900112
block_4399_3821_4339_ch1.tif	0.98431	0.972624	0.978432	0.98091	27.60435	0.957775
block_4428_5129_3259_ch1.tif	0.977812	0.991369	0.984544	0.985715	26.07681	0.969558
block_4603_5384_4879_ch1.tif	0.945974	0.953139	0.949543	0.973811	24.73863	0.903933
block_4787_3606_4079_ch1.tif	0.991558	0.973044	0.982214	0.972535	40.42277	0.965049
block_5041_3791_8479_ch1.tif	0.993648	0.878115	0.932316	0.976353	41.24318	0.873213
block_5089_4849_5239_ch1.tif	0.971276	0.979338	0.97529	0.98394	9.899495	0.951772
block_5207_4811_3259_ch1.tif	0.983139	0.976343	0.979729	0.993849	47.21229	0.960264
block_5582_3923_3799_ch1.tif	0.997855	0.97299	0.985266	0.996484	45.04442	0.970959
block_5981_4531_2539_ch1.tif	0.997742	0.882819	0.936769	0.984504	22.02272	0.881059
block_5997_5939_6319_ch1.tif	0.99656	0.968412	0.982284	0.978959	9.273618	0.965186
block_6009_5240_3539_ch1.tif	0.926206	0.965119	0.945262	0.961087	19.72308	0.896205
block_6083_2958_3619_ch1.tif	0.990036	0.93833	0.96349	0.958603	21.56386	0.929552
block_6084_2665_6679_ch1.tif	0.96891	0.97897	0.973914	0.9675	35.69314	0.949154
block_6463_4389_4259_ch1.tif	0.97256	0.983739	0.978117	0.979546	26.24881	0.957172
block_6619_4151_4619_ch1.tif	0.997201	0.903462	0.94802	0.989292	7	0.901177
block_7547_3960_6599_ch1.tif	0.994983	0.938149	0.96573	0.974163	49.09175	0.933732
block_7708_3900_5159_ch1.tif	0.995712	0.936409	0.96515	0.97178	33.74907	0.932648
block_7751_5462_5519_ch1.tif	0.994663	0.912171	0.951632	0.989533	5.196152	0.907728
block_7882_5182_5519_ch1.tif	0.997988	0.974079	0.985888	0.989515	21.2132	0.972169
**Average**	**0.984305**	**0.948421**	**0.965482**	**0.977053**	**28.88697**	**0.933774**

Table S5. The performance of V-Net on test data.

Cases	Precision	Recall	Dice	clDice	Hausdorff	Jaccard
block_3044_4250_9739_ch1.tif	0.995551	0.919382	0.955951	0.981336	39.8748	0.91562
block_3782_3113_3259_ch1.tif	0.977479	0.971822	0.974642	0.872914	42.55585	0.950539
block_3806_5055_4879_ch1.tif	0.995158	0.905369	0.948143	0.987935	67.14909	0.901399
block_4399_3821_4339_ch1.tif	0.970998	0.990008	0.980411	0.961702	27.60435	0.961575
block_4428_5129_3259_ch1.tif	0.969312	0.995642	0.9823	0.989388	28.44293	0.965216
block_4603_5384_4879_ch1.tif	0.940189	0.959721	0.949854	0.96043	26.98148	0.904498
block_4787_3606_4079_ch1.tif	0.975461	0.97785	0.976654	0.963736	37.73592	0.954373
block_5041_3791_8479_ch1.tif	0.979366	0.900509	0.938283	0.97568	14.86607	0.883742
block_5089_4849_5239_ch1.tif	0.968996	0.980519	0.974724	0.973041	11.83216	0.950693
block_5207_4811_3259_ch1.tif	0.979754	0.975846	0.977796	0.978912	46.62617	0.956557
block_5582_3923_3799_ch1.tif	0.9951	0.97216	0.983496	0.993618	45.04442	0.967528
block_5981_4531_2539_ch1.tif	0.992723	0.882727	0.934499	0.981646	29.5804	0.877051
block_5997_5939_6319_ch1.tif	0.99442	0.9752	0.984716	0.982715	123.065	0.969893
block_6009_5240_3539_ch1.tif	0.925387	0.993647	0.958303	0.969149	39.59798	0.919944
block_6083_2958_3619_ch1.tif	0.978892	0.919026	0.948015	0.924434	19.46792	0.901168
block_6084_2665_6679_ch1.tif	0.957359	0.989523	0.973175	0.964245	35.69314	0.947752
block_6463_4389_4259_ch1.tif	0.955902	0.98368	0.969592	0.929836	33.24154	0.940979
block_6619_4151_4619_ch1.tif	0.993723	0.897721	0.943286	0.978569	61.10646	0.89266
block_7547_3960_6599_ch1.tif	0.991699	0.940546	0.965446	0.971009	49.09175	0.9332
block_7708_3900_5159_ch1.tif	0.983835	0.94091	0.961894	0.969487	33.97058	0.926586
block_7751_5462_5519_ch1.tif	0.97904	0.913026	0.944881	0.96917	29.27456	0.895521
block_7882_5182_5519_ch1.tif	0.993278	0.972887	0.982977	0.980579	41.04875	0.966524
**Average**	**0.976983**	**0.952624**	**0.964047**	**0.966342**	**40.17506**	**0.931046**

Table S6. The performance of FCNs on test data.

Cases	Precision	Recall	Dice	clDice	Hausdorff	Jaccard
block_3044_4250_9739_ch1.tif	0.995148	0.916397	0.95415	0.98821	39.31921	0.912321
block_3782_3113_3259_ch1.tif	0.976663	0.997036	0.986744	0.978003	42.16634	0.973835
block_3806_5055_4879_ch1.tif	0.996328	0.887116	0.938556	0.990708	18.92089	0.884225
block_4399_3821_4339_ch1.tif	0.976528	0.985698	0.981092	0.967093	27.60435	0.962885
block_4428_5129_3259_ch1.tif	0.987474	0.986831	0.987152	0.990104	27.65863	0.974631
block_4603_5384_4879_ch1.tif	0.936598	0.977939	0.956822	0.986536	23.87467	0.917218
block_4787_3606_4079_ch1.tif	0.974463	0.967558	0.970998	0.97246	37.73592	0.943631
block_5041_3791_8479_ch1.tif	0.976877	0.884287	0.928279	0.980697	41.24318	0.866157
block_5089_4849_5239_ch1.tif	0.969418	0.985946	0.977612	0.989917	11.7047	0.956204
block_5207_4811_3259_ch1.tif	0.9825	0.975459	0.978967	0.987361	55.98214	0.9588
block_5582_3923_3799_ch1.tif	0.9966	0.960871	0.97841	0.995887	45.04442	0.957732
block_5981_4531_2539_ch1.tif	0.996807	0.863525	0.925392	0.983528	22.04541	0.861143
block_5997_5939_6319_ch1.tif	0.995887	0.968602	0.982055	0.992087	2.236068	0.964743
block_6009_5240_3539_ch1.tif	0.930452	0.997147	0.962646	0.986915	13.0384	0.927981
block_6083_2958_3619_ch1.tif	0.982238	0.967813	0.974972	0.976203	23.34524	0.951167
block_6084_2665_6679_ch1.tif	0.953281	0.991753	0.972137	0.966203	35.69314	0.945784
block_6463_4389_4259_ch1.tif	0.986555	0.982883	0.984716	0.934489	32.52691	0.969891
block_6619_4151_4619_ch1.tif	0.993372	0.907935	0.948734	0.985025	35.86084	0.902468
block_7547_3960_6599_ch1.tif	0.994732	0.935312	0.964107	0.987887	49.09175	0.930701
block_7708_3900_5159_ch1.tif	0.988212	0.947896	0.967634	0.983022	33.97058	0.937298
block_7751_5462_5519_ch1.tif	0.993035	0.903812	0.946325	0.985465	31.38471	0.898119
block_7882_5182_5519_ch1.tif	0.990298	0.976897	0.983552	0.990414	41.04875	0.967636
**Average**	**0.980612**	**0.953123**	**0.965957**	**0.981737**	**31.43165**	**0.934753**

Table S7. The performance of MPL on test data.

Cases	Precision	Recall	Dice	clDice	Hausdorff	Jaccard
block_3044_4250_9739_ch1.tif	0.947023	0.986456	0.966338	0.991202	39.9124	0.934868
block_3782_3113_3259_ch1.tif	0.974194	0.994642	0.984312	0.985242	42.16634	0.969108
block_3806_5055_4879_ch1.tif	0.986804	0.938286	0.961933	0.990863	5.385165	0.926659
block_4399_3821_4339_ch1.tif	0.9594	0.986103	0.972568	0.975456	30.64311	0.946602
block_4428_5129_3259_ch1.tif	0.988286	0.958393	0.97311	0.991311	26.07681	0.947627
block_4603_5384_4879_ch1.tif	0.934105	0.963986	0.94881	0.985677	23.87467	0.902606
block_4787_3606_4079_ch1.tif	0.98259	0.956313	0.969274	0.986848	40.42277	0.940379
block_5041_3791_8479_ch1.tif	0.97457	0.935632	0.954704	0.993094	5	0.913334
block_5089_4849_5239_ch1.tif	0.957239	0.982449	0.96968	0.995077	8.774964	0.941145
block_5207_4811_3259_ch1.tif	0.978211	0.969421	0.973796	0.994952	47.20169	0.94893
block_5582_3923_3799_ch1.tif	0.985008	0.965605	0.97521	0.997415	45.04442	0.951619
block_5981_4531_2539_ch1.tif	0.97075	0.954782	0.9627	0.991735	22.04541	0.928082
block_5997_5939_6319_ch1.tif	0.97042	0.98121	0.975785	0.987487	104.9667	0.952716
block_6009_5240_3539_ch1.tif	0.925055	0.993434	0.958026	0.98772	8.062258	0.919433
block_6083_2958_3619_ch1.tif	0.970083	0.957801	0.963903	0.983334	23.36664	0.930321
block_6084_2665_6679_ch1.tif	0.964355	0.975391	0.969842	0.986013	35.60899	0.94145
block_6463_4389_4259_ch1.tif	0.993231	0.970642	0.981807	0.993558	14.28286	0.964264
block_6619_4151_4619_ch1.tif	0.972788	0.935477	0.953768	0.990325	8	0.911621
block_7547_3960_6599_ch1.tif	0.988397	0.94526	0.966347	0.995038	49.09175	0.934885
block_7708_3900_5159_ch1.tif	0.949839	0.977999	0.963713	0.986907	29.42788	0.929968
block_7751_5462_5519_ch1.tif	0.978193	0.931054	0.954042	0.99456	4.690416	0.912122
block_7882_5182_5519_ch1.tif	0.988076	0.964827	0.976313	0.993547	5	0.953722
**Average**	**0.969937**	**0.96478**	**0.96709**	**0.98988**	**28.13842**	**0.93643**

Table S8. The performance times of HP-VSP on a desktop and a high-performance computer. Units: hours.

	Desktop	HPC
Blocking	2.06	0.18
Predicting	7.72	1.82
Fusion	11.83	0.72
Total time	21.62	2.72

Table S9. Quantification of the vessel length density in the three samples (six hemispheres). Units are in mm/mm^3.

**Sample**	**CA1-2**	**CA3**	**DG**	**ALL**
#193882_l	517.3881574	566.7869	581.9384	548.2855
#193882_r	508.1386905	523.0461	568.8738	528.8786
#194776_l	535.4813654	560.6583	657.3062	575.8353
#194776_r	531.3606841	551.5286	654.0066	570.62
#201620_l	498.339616	541.3164	695.8754	564.3406
#201620_r	538.4263942	553.153	664.9566	577.3247
**Average**	**521.5224846**	**549.4149**	**637.1595**	**560.8808**

Table S10. Quantification of the branch point density in the three samples (six hemispheres). Units are in $count/mm^3$.

**Sample**	**CA1-2**	**CA3**	**DG**	**ALL**
#193882_l	4600.110035	5172.296136	5371.880832	4964.65837
#193882_r	4532.146989	4593.695536	5186.054738	4729.331377
#194776_l	4808.43593	4844.375651	6340.185455	5241.769802
#194776_r	4791.092244	4713.418112	6121.546825	5138.744756
#201620_l	4821.7462	5221.449099	7920.544314	5784.689558
#201620_r	5484.436317	5484.768547	7355.030307	6002.135405
**Average**	**4839.661286**	**5005.000514**	**6382.540412**	**5310.221544**

Table S11. Quantification of the radius distribution of vessels in the hippocampal region.

**Radius**	**1**	**2**	**3**	**4**	**5**	**6**	**7**	**8**	**9**	**>10**
**Percentage(%)**	8.78	56.08	21.10	5.27	3.40	1.42	0.88	0.82	0.43	1.82
